# Cast Iron Heating Devices

**Published:** 1875-12

**Authors:** 


### CAST-IRON HEATING DEVICES.

There is a singular error extant concerning the porosity of cast-iron furnaces and stoves. It has been published hundreds of times, that the gases of combustion escape readily through a cast-iron fire-pot, and that these gases cannot get through a fire-pot made of wrought iron. This is clearly a fallacy. Coal-gas will find its way through the one just as readily as through the other. It cannot get through either unless the iron has a flaw, a crack, or a hole through it. Both kinds of iron, if of good quality, are as impermeable as glass. This is shown by the reports of Fremy, Payen, DeVille and Morin, of Paris, to the French Academy of Sciences, after intricate and exhanstive experiments lasting more than a year. They decided that while the air surrounding a furnace or stove could be impaired, provided the iron were raised to a red heat, the deterioration would be just as great when the fuel was consumed in wrought-iron as when consumed in cast-iron. Both are impervious and impermeable by gas of any kind.

The fact is, the harm done to this atmosphere, heated by means of an iron stove or furnace, need not occur if the fire-pot were properly lined with fire-brick, so that the iron could never become red-hot. Iron of any kind, heated to redness, and exposed to air, generates an unbreatheable gas called carbonic oxide. This will be the result, whether the fuel be anthracite, or charcoal, or wood, or coke. People who breathe air warmed by a red-hot stove or furnace, will suffer and die, die slowly, perhaps, but surely. This is where the advantage comes in of the old-fasioned open fire-place. There is no red-hot iron surfaces to de-vitalize and poison the atmosphere. But as we cannot all keep warm by fire-places, let us see if we cannot heat our dwellings comfortably by the use of large stoves and furnaces, so that there may be no need of forcing them up to the destructive redness which takes the life out of the air and adds to it a noxious exhalation.

Mr. A. M. Lesley's Gothic Furnace, which we examined so critically last year, appears to be the safest and most durable hot-air furnace in use. Its large fire-pot and immense, dome-like radiating surface, render heating to redness altogether unnecessary. The air of the house heated by this furnace seems pure and pleasant to breathe. We copy a recent letter which refers especially to its capacity as a heater:

*Alex. M. Lesley*, 226 *West* 23*d st., N. Y.*

Dear-sir: Something over eleven years ago I had one of your No. 10 Gothic Furnaces placed in my house, situated on the high ground in State St. (near the Capitol) and considerably exposed to a cold climate in winter. I have depended upon it as the principal means of heating my house, which is 25x60 and four stories above the basement. One side of the house is disconnected from any building.

Your furnace has done much more than I expected. I have had no repairs except a grate, at a trifling cost.

Truly yours, Geo, S. Weaver.

162 State Street, Albany, N. Y.

